# Humanoids for teaching and training coronary artery bypass surgery to the next generation of cardiac surgeons

**DOI:** 10.1093/icvts/ivab260

**Published:** 2021-10-12

**Authors:** Piergiorgio Tozzi, Enrico Ferrari, Oliver Reuthebuch, Peter Matt, Christoph Huber, Friedrich Eckstein, Matthias Kirsch, Carlos A Mestres

**Affiliations:** 1 Cardiac Surgery, Lausanne University Hospital, Centre Hospitalier Universitaire Vaudois, Lausanne, Switzerland; 2 Cardiac Surgery, Cardiocentro Ticino Institute, Lugano, Switzerland; 3 Cardiac Surgery, Basel University Hospital, Basel, Switzerland; 4 Cardiac Surgery, Cantonal Hospital, Lucerne, Switzerland; 5 Cardio-Vascular Department, Geneva University Hospital, Geneva, Switzerland; 6 Cardiac Surgery, University Hospital Zürich, Zürich, Switzerland

**Keywords:** Coronary artery bypass grafting, Surgical simulator, Training

## Abstract

**OBJECTIVES:**

Technical skills are an essential component of cardiac surgery, and the operating room is becoming an even more challenging environment for trainees who want to acquire such skills. Simulation, which partially overcomes this limitation, represents a valid adjunct to surgical education. We describe an original simulator and provide results in terms of trainees’ evaluations and ratings.

**METHODS:**

We used a humanoid that is a silicone replica of the chest of an adult human that provides a complete anatomical platform for hands-on skin-to-skin practice of surgical techniques in arrested heart coronary artery bypass graft (CABG) surgery cases. Learners were residents in cardiac surgery. The teaching sessions included 2 full three-vessel CABG procedures using both mammary arteries and a hydrogel vein. Five board-certified cardiovascular surgeons scored the surgical activity of all trainees. The trainees were asked to complete an exit questionnaire to evaluate their course.

**RESULTS:**

Overall, 16 residents participated in the simulation, including 5 women and 11 men, with a mean age of 30 ± 4 years, all of whom had at least 2 years of cardiac surgery training. All participants completed the 2 CABG operations. Three mammary arteries (4.6%) were seriously damaged during harvesting. In 1 case (3.1%), an aortic tear occurred during aortic cannulation. Each trainee performed overall 6 distal and 2 proximal coronary anastomoses. All participants agreed that the ‘humanoid reproduces real-life situations, the feeling is realistic, and they are now more confident in performing coronary anastomosis’.

**CONCLUSIONS:**

Trainees involved in this simulation curriculum acquired and refined technical skills that could be applied directly to human patients. In addition, we were able to foster a higher level of teamwork within the operating room team.

**Figure:**
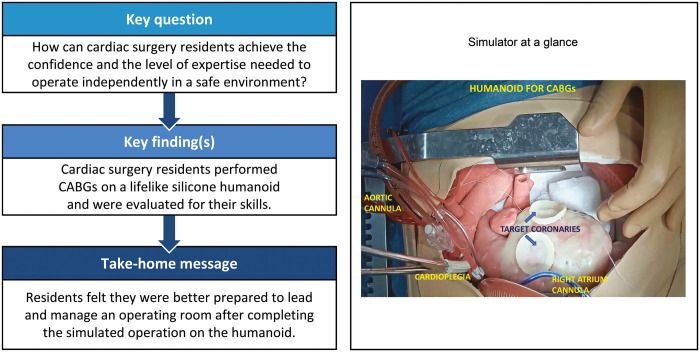

## INTRODUCTION

Technical skills are an essential component of cardiac surgery. The operating room has become an even more challenging environment in which to acquire such skills. The apprentice model of operating room teaching exclusively has substantially lost its efficacy, given that this model provides insufficient time for technical skills to be taught due to work-hour restrictions and supervision rules [1]. Moreover, the apprentice model in humans is associated with a low tolerance for learning inefficiency, eliminates deliberate practice and does not ensure enough exposure to rare yet grave undesirable events [2]. Consequently, this teaching model alone does not provide residents with adequate operating experience that would enable them to acquire the confidence and level of expertise needed to operate independently. Simulation should help partially overcome current limitations of the apprentice model while being an adjunct to cardiac surgery education. It should thus be considered as an educational technique that allows trainees to mimic real-world experiences in an interactive manner [3].

The idea of acquiring complex skills with potential harmful complications by simulating the activity within a safe environment is an old one. In 1929, the first simulator was developed for training aviators. Since then, using a simulator in aircraft training has been consistently found to produce improvements in take-offs, approaches and landings while definitely improving the safeness of air travel [4]. In surgery, simulation was introduced at the end of the 20th century [5] while relying on different fidelity-to-reality levels, the extremes being the low-fidelity and high-fidelity simulators. The low-fidelity systems have been developed for training a single task that is part of a more complex procedure, such as learning how to construct a coronary anastomosis. Such systems, which are particularly appreciated by beginners, have the advantage of being relatively inexpensive, yet easily accessible. At the opposite end are the high-fidelity devices like the Ramphal simulator [6], which enables the training of complex surgical procedures. Such devices are extremely expensive and available only in qualified training centres. Recent studies have demonstrated that the degree of satisfaction and the usefulness for residents and faculty members involved as tutors in the simulation were directly proportioned to the complexity of the simulation. In other words, the more realistic the simulation, the more it was appreciated by the users [2]. The most advanced and realistic simulator for cardiac surgery is probably the Ramphal simulator [6], but it has the inconvenience of using animal organs and is therefore associated with logistic and sanitary restrictions.

The Swiss Society for Cardiac and Thoracic Vascular Surgery (SGHC) has decided to raise the Swiss educational and training standards by creating a Swiss Academy for training cardiac surgeons on lifelike simulators. In an effort to improve both surgical skills and competences in handling operative complications (simulation-based curriculum), our goal was to integrate courses on lifelike simulators, also called humanoids, into the standard cardiac surgery training programme (clinical curriculum). To accomplish our goal of developing and running a high-fidelity curriculum on coronary artery bypass grafting (CABG), we created a committee that included at least 1 representative from each Swiss training hospital. The overall committee objectives were as follows:

- Provide Swiss cardiac surgery residents with the extremely valuable opportunity to learn step by step the entire surgical procedure of CABG;
- Contribute to an increase in educational teaching standards by applying standards of practice defined by the SGHC.

Our goal was to present our original teaching tool and our early results in terms of residents’ evaluation and rating.

## METHODS

### Ethical statement

There was no need for ethics committee approval to carry out this study.

### Study design

This study was designed as a prospective evaluation of a high-fidelity humanoid skin-to-skin CABG training model for surgical trainees. The term humanoid refers to an artificial structure that has a physical form similar to that of a human [7].

### The humanoid

**Figure 1:**
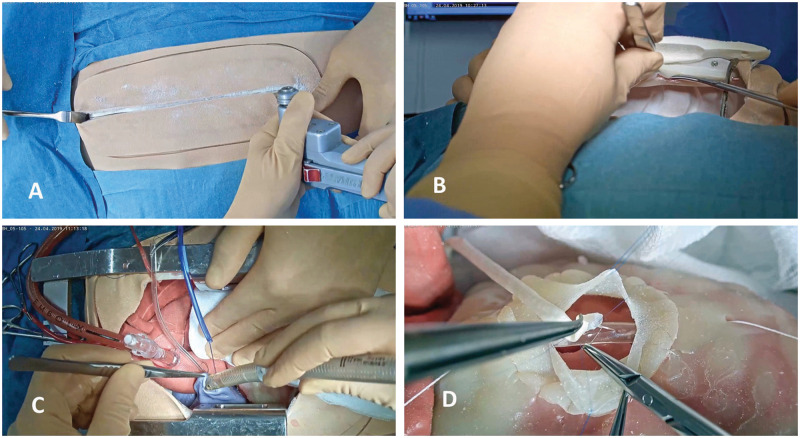
Synopsis of the different learning pathways that the humanoid provides. (**A**) Skin incision and sternotomy. (**B**) Left internal mammary artery harvesting with the possibility of checking patency and leaks after the takedown. (**C**) Insertion of aortic and right atrium cannulas for cardiopulmonary bypass connection. Insertion of the cardioplegia needle. The trainee had to control possible bleeding from the aortic and the right atrium access. (**D**) Coronary anastomosis construction after having dissected the coronary artery from the epicardium.

In collaboration with a member of the professional simulation industry (The Chamberlain Group, Great Barrington, MA, USA), we developed a cardiothoracic surgical simulator designed to practice training strategies associated with surgical revascularization of patients with coronary artery disease. This humanoid was a 1:1 silicone replica of an adult human chest that provided a complete anatomical platform for the hands-on skin-to-skin practice of surgical techniques for arrested heart CABG. Given this context, the humanoid developed by the trainers enabled the trainees to perform a sternotomy, including identifying landmarks by tactile feel, incising the skin, cutting the sternum and wiring it closed at the end of the procedure. The model included replaceable high-fidelity right internal mammary arteries (IMAs) and left internal mammary arteries (LIMAs), along with their side branches attached to the underside of the sternal ribs to allow the trainees to practice their takedown, check their patency and look for artery wall lesions due to harvesting (Fig. 1). The ascending aorta and right atrium enabled the insertion of cardiopulmonary bypass cannulas; the aorta was configured to allow for cross-clamping. Replaceable epicardial discs on the surface of the heart must be incised for the target coronary arteries to be exposed when performing CABG (Fig. 1). Its modular chambers and aorta are replaceable and easily exchanged for deliberate practice without using any animal tissues. An open hydraulic circuit enabled aortic and right atrial perfusion to reproduce physiologic pressures, consisting of 90 mmHg in the aorta and 5 mmHg in the right atrium, in order to render the cannulation procedure more realistic and to handle bleeding complications at the cannulation sites. The integrity of the harvested IMAs, in addition to the patency and sealing of the anastomosis, was assessed by injecting them with artificial blood.

### Simulation set-up

**Figure 2:**
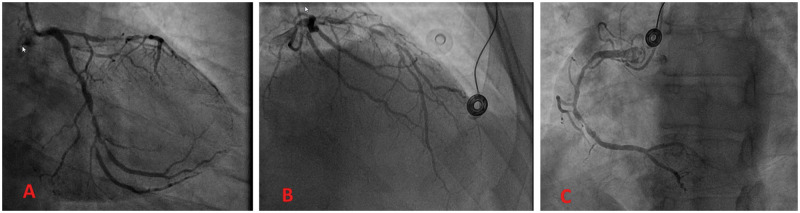
Simulated patient coronary angiogram to define the revascularization strategy. (**A**) Right oblique anterior view showing 70–90% stenosis of the obtuse marginal. (**B**) Right oblique anterior caudal view showing subocclusion of the proximal left anterior descending. (**C**) Left oblique anterior view showing 70–90% stenosis of the distal right coronary artery.

**Figure 3:**
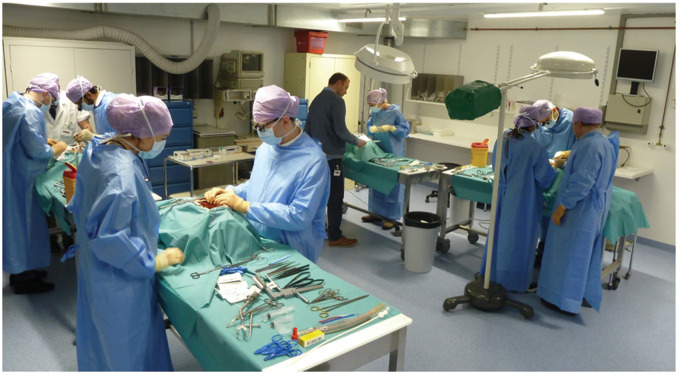
Overview of the teaching set-up. Four fully equipped working stations enabled 2 trainees at each station to perform the entire coronary artery bypass procedure.

The clinical set-up simulated the treatment of a 60-year-old patient with three-vessel disease (Fig. 2). Our learners were cardiac surgery trainees from 5 Swiss university hospitals; their training was conducted over 2 consecutive days. The teaching sessions included a 4-h preliminary training session to perform coronary anastomoses on a simplified model, which was followed by performing twice a full three-vessel CABG procedure using both IMAs and 1 hydrogel vein. The procedures were facilitated by 2 certified cardiac surgeons with more than 20 years of practice who supervised both the exercises and the support staff (Fig. 3).

**Figure 4:**
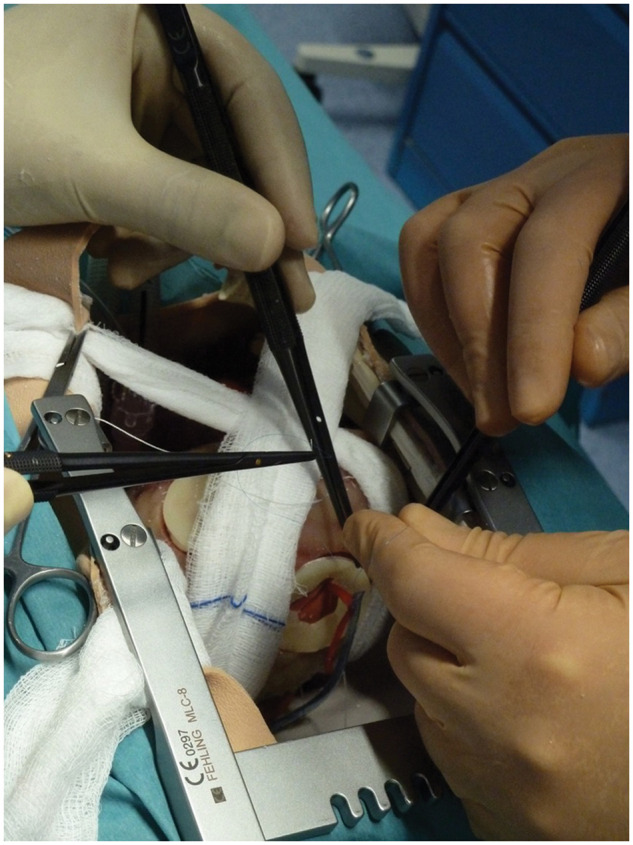
Residents learned specific tasks, such as how to expose the obtuse marginal artery. A first wet sponge is passed through the transverse sinus and anchored to the left arm of the sternal retractor; a second wet sponge is inserted between the inferior vena cava and inferior right pulmonary vein and anchored to the upper part of the sternal retractor’s right arm.

The clinical case was discussed extensively beforehand in order to better define the revascularization strategy and conduit options (LIMA on left anterior descending; right internal mammary artery as a Y graft on LIMA or passed through the transverse sinus to reach the obtuse marginal branch; vein graft to the right coronary artery or obtuse marginal branch). Surgical target vessel exposure was likewise presented and discussed prior to starting the procedures (Fig. 4). Notably, all surgical instruments, including microsurgery instruments, were brand new.

### Trainee assessment

The Objective Structured Assessment of Technical Skills (OSATS) is the most precise and widely used approach to assessing trainees’ surgical performance within a simulated environment [8]. OSATS parameters, which are illustrated in Table 1, were applied to evaluate the procedure’s key steps except for construction of the anastomosis. To that end, we applied the OSATS dedicated to evaluating a coronary anastomosis, as described by Fann *et al.* [9] (Table 2). According to this OSATS, the procedure of performing a coronary anastomosis is broken down into 9 teachable components, with a conceptual, virtual and operational learning process identified for each teachable component. Each teachable component of the OSATS can be scored as 1 for poor; 2 for below average; 3 for average; 4 for good; and 5 for excellent.

**Table 1:** OSATS: Objective Structured Assessment of Technical Skills (adapted from Martin *et al.*, 1997)

	UNDER LEVEL 1	2	ON LEVEL 3	4	ABOVE LEVEL 5
Respect of tissue	Frequently used unnecessary force on tissue or caused damage by inappropriate use of instruments		Careful handling of tissue but occasionally caused inadvertent damage		Consistently handled tissues appropriately with minimal damage
Time and motion	Many unnecessary movements		Efficient time/motion but some unnecessary movements		Clear economy of movement and maximum efficiency
Instrument handling	Repeatedly makes tentative or awkward moves with instruments by inappropriate use of instruments		Competent use of instruments but occasionally appeared stiff or awkward		Fluid moves with instruments and no awkwardness
Knowledge of instruments	Frequently asked for wrong instrument or used inappropriate instrument		Knew names of most instruments and used appropriate instruments for the task		Obviously familiar with the instruments and their names
Flow of operation	Frequently stopped operating and seemed unsure of the next movement		Demonstrated some forward planning with reasonable progression of procedure		Obviously planned course of operation with effortless flow from 1 move to the next
Use of assistants	Consistently placed assistants poorly or failed to use assistants		Appropriate use of assistants most of the time		Strategically used assistants to the best advantage at all times
Knowledge of specific procedure	Deficient knowledge. Needed specific instruction at most steps		Knew all important steps of the operation		Demonstrated familiarity with all aspects of the operation

**Table 2:** Teachable components of performance rating scores for coronary anastomoses (adapted from Fann *et al*., 2008)

1. Graft orientation (proper orientation for toe–heel, appropriate start and end points)	1	2	3	4	5
2. Bite appropriate (entry and exit points, number of punctures, even and consistent distance from edge)	1	2	3	4	5
3. Spacing appropriate (even spacing, consistent distance from previous bite, too close vs too far)	1	2	3	4	5
4. Use of Castroviejo needle holder (finger placement, instrument rotation, facility, needle placement, pronation and supination, proper finger and hand motion, lack of wrist motion)	1	2	3	4	5
5. Use of forceps (facility, hand motion, assist needle placement, appropriate traction on tissue)	1	2	3	4	5
6. Needle angles (proper angle relative to tissue and needle holder, consider depth of field, anticipating subsequent angles)	1	2	3	4	5
7. Needle transfer (needle placement and preparation from stitch to stitch, use of instrument and hand to mount needle)	1	2	3	4	5
8. Suture management/tension (too loose vs too tight, use tension to assist exposure, avoid entanglement)	1	2	3	4	5
9. Knot tying (adequate tension, facility, and finger and hand follow-up for deep knots)	1	2	3	4	5

Scores: 1, excellent, able to accomplish goal without hesitation, showing excellent progress and flow; 2, good, able to accomplish goal deliberately, with minimal hesitation, showing good progress and flow; 3, average, able to accomplish goal with hesitation, discontinuous progress and flow; 4, below average, able to partially accomplish goal with hesitation; 5, poor, unable to accomplish goal, marked hesitation.

Five board-certified surgeons from the SGHC scored the surgical activity of all trainees in their turn, with the final score then presented to each trainee in a 1-to-1 interview at the end of the second day’s training session.

### Curriculum assessment

The curriculum was evaluated by the supervisors according to different perspectives in real time throughout the simulation, as well as afterwards in post-training qualitative interviews with all participants.

### Trainee rating using an exit questionnaire

After receiving their evaluation, the participants were asked to complete an exit questionnaire consisting of 6 statement scores as follows: ‘agree’; ‘somewhat agree’; ‘not sure’; ‘somewhat disagree’; and ‘disagree’. The questionnaire’s purpose was to assess the trainees’ opinions on the realism of the simulation tasks, efficacy of the simulator training experience and their own confidence in performing the surgical procedures following simulator training (Table 3).

**Table 3:** Exit questionnaire administered to trainees at the end of the course

1. The humanoid reproduces real-life situations including team approach
2. From skin incision to cannulation, the simulator reproduces a realistic feeling
3. Performing an anastomosis on the humanoid model is a realistic representation of the procedure
4. The humanoid is a good method of training technical skills
5. The humanoid is a good method of learning the handling of procedure complications
6. I am more confident in coronary anastomosis

## RESULTS

### Trainee population

Overall, 16 residents in cardiac surgery from 5 Swiss University Hospitals participated in the study, including 5 women and 11 men, with a mean age and standard deviation of 30 ± 4 years. Their cardiac surgery training ranged from 2 to 4 years, with a mean of 2.8 years; they had participated in at least 100 cardiac interventions as assistants.

### Operation completion

All participants completed the 2 assigned CABG operations in 240 ± 60 min for each procedure. Three IMAs, which comprised 4.6% of all harvested IMAs, were damaged during harvesting and thus were not used for the bypass. In 1 case (3.1% of aortic cannulations), an aortic tear occurred during aortic cannulation. Each trainee performed overall 6 distal (left anterior descending, obtuse marginal and right coronary artery at its bifurcation) and 2 proximal anastomoses on the ascending aorta, using 8/0 and 6/0 running polypropylene sutures. Median time for constructing the distal anastomosis was 18.5 min (range 12–31 min).

### Exit questionnaire

All participants filled out the exit questionnaire and agreed on the point that simulation reproduces real-life situations, that it produces a realistic feeling and that the trainees were actually more confident in performing a coronary anastomosis. Overall, 12/16 (75%) agreed that ‘performing an anastomosis on the humanoid model is a realistic procedure representation’, whereas 11/16 (68%) agreed that ‘the humanoid helps learn how to handle surgical complications’.

## DISCUSSION

The literature suggests that surgical expertise is reached through practice, meaning that, commonly, surgical experts are made, not born [10]. The challenge for the surgical educator is to develop training programmes that can address shortfalls for every trainee, regardless of their ability.

Successful achievement of any surgical procedure is dependent, though not exclusively, on the acquisition and execution of psychomotor skills. Based on a three-phase theory, there is first a cognitive phase, during which the skill is understood and practiced, allowing trainees to make errors; this is followed by an integrative phase when performance becomes more fluent; finally, there is an autonomous phase when the motor skills are being carried out without much conscious effort. In any case, deliberate practice has been addressed in the past as a pathway to attain the needed expertise [11].

With given work-hour limitations and increasing concerns about patient outcomes [1, 2], it has become necessary to be innovative with educational methods in an effort to improve residents’ knowledge and skills. This goal specifically comprises increasing innovation in teaching platforms and curricula. Our project’s ultimate goal was to develop and provide a high-fidelity realistic simulator for cardiac surgery residents to let them directly practice all of the steps involved in on-pump CABG.

Traditionally, in the cardiac simulation field, much of the emphasis has been focused on singular tasks, such as the proper creation of a coronary anastomosis, without reproducing the complexity and challenges of the entire procedure. This is a major limitation of such simulators, and in particular for advanced trainees, given that this method skips the preliminary and fundamental steps that are common to all cardiac surgery procedures.

Swiss cardiac surgeons have a long track record in terms of CABG simulation. In 2002, Reuthebuch *et al.* [12] published a paper on the Zurich heart-trainer, which was the first advanced training model for beating-heart coronary artery surgery. The similarity to human tissue and the easy set-up made this completely artificial model an ideal teaching tool designed to increase the confidence of cardiac surgeons dealing with the beating heart and applying minimally invasive surgery.

We strongly believe that the model presented herewith deserves the definition of humanoid, even though this term does not actually exist in academic simulation literature. This model allows the trainee to reproduce each step of a real CABG procedure based on the following sequence: starting from the skin incision based on bone landmarks to sewing the sternum, harvesting the right internal mammary artery and LIMA, opening the pericardium, connecting the cardiopulmonary bypass components, cross-clamping the aorta, injecting the cardioplegia, identifying the target vessel through the epicardium, carrying out the coronary anastomosis, checking the anastomosis patency, performing the proximal anastomosis, removing the cardiopulmonary bypass components and eventually closing both the sternum and skin.

The model enabled the residents to learn specific tasks, such as how correctly to expose the targeted vessels, particularly the obtuse marginal and right coronary arteries, using slings and stitches (Fig. 4).

The use of new surgical instruments played a key role in the teaching process. Owing to budget restrictions, residents were invited to use, most of the time, broken or faulty instruments. This rendered the surgical activity more complex and even frustrating, which dramatically decreased the trainees’ concentration and enthusiasm. In contrast, using perfectly working surgical instruments facilitated the learning process, which was greatly appreciated by the participants.

In addition, the residents greatly appreciated the possibility to learn different strategies for handling complications, such as adding an extra stitch on the anastomosis or controlling the bleeding on cannulation sites in a safe and stressless environment.

Learning surgery using lifelike simulators also reduces the need to train on live animals, which is in full compliance with recent laws for protecting laboratory animals according to the ‘three Rs’ concept [13]. In our view, humanoids like the one described in this manuscript will, with time, render the use of animals completely unnecessary.

The residents clearly highlighted the acquisition of specific targeted surgical skills and their increased proficiency in these. These newly acquired or further practiced skills were felt to be directly transferable to the real operating room environment. In addition, the residents were similarly appreciative of the opportunity to practice various roles including those of communicator, collaborator and manager. The simulation set-up provided them the opportunity to demonstrate leadership skills within the operating room, which turned out to be a key component of their growth and development. This setting also afforded the senior residents the opportunity to practice their teaching and guidance skills on their junior colleagues in a safe environment. Furthermore, during the simulation, communication among team members and the briefings before and after the sessions significantly contributed to a real team approach feeling.

A heart simulator with an integrated supervision system could enable the trainee to practice surgery without constant teacher feedback. We are in the process of developing a mitral surgery simulator with integrated sensors. This system should be able to generate, record and display quantitative data concerning trainee performance in regard to the mitral valve repair procedure. This integrated supervision system heart-surgery simulator could offer a real-life model for learning about and training in mitral valve surgery, providing both immediate and precise feedback about the accuracy of suture placement. This feature could potentially replace the teaching role of the experienced surgeon. We are also planning to use simulators for aortic valve and thoracic aorta surgery; these simulators are being developed by Vascular International Inc., Switzerland (https://vascular-international.org).

### Limitations

Our study has several limitations that deserve to be mentioned. Our trainee population was limited to 16 participants, which is relatively small. In addition, we did not apply any statistical analyses to assess the statistical significance of the results. With respect to overall costs, the cost per simulator amounted to 600 US$ plus 100 US$ for ancillary material.

## CONCLUSIONS

The humanoid that we presented reproduced real-life situations, and the residents participating in this simulation curriculum successfully acquired and refined technical skills that could be directly extrapolated to humans. According to the learners, they actually felt better equipped to lead and manage an operating room after completing the simulation. Moreover, they were able to foster a higher level of teamwork within the operating room team.

## ABBREVIATIONS

CABG: Coronary artery bypass graft
IMAs: Internal mammary arteries
LIMAs: Left internal mammary arteries
OSATS: Objective Structured Assessment of Technical Skills
SGHC: Society for Cardiac and Thoracic Vascular Surgery
